# Methodologies to generate, extract, purify and fractionate yeast ECM for analytical use in proteomics and glycomics

**DOI:** 10.1186/s12866-014-0244-0

**Published:** 2014-10-25

**Authors:** Fábio Faria-Oliveira, Joana Carvalho, Celso LR Belmiro, Montserrat Martinez-Gomariz, Maria Luisa Hernaez, Mauro Pavão, Concha Gil, Cândida Lucas, Célia Ferreira

**Affiliations:** CBMA (Centre of Molecular and Environmental Biology), Department of Biology, University of Minho, Braga, Portugal; Institute of Medical Biochemistry, Laboratory of Glycoconjugates Biochemistry and Cellular Biology, Federal University of Rio de Janeiro/ Polo de Macaé, Macaé, Brazil; Unidad de Proteómica, Universidad Complutense de Madrid – Parque Científico de Madrid UCM-PCM), Madrid, Spain; Institute of Medical Biochemistry, Laboratory of Glycoconjugates Biochemistry and Cellular Biology, Federal University of Rio de Janeiro, Rio de Janeiro, Brazil; Departamento de Microbiología II, Facultad de Farmacia, Universidad Complutense de Madrid, Madrid, Spain

## Abstract

**Background:**

In a multicellular organism, the extracellular matrix (ECM) provides a cell-supporting scaffold and helps maintaining the biophysical integrity of tissues and organs. At the same time it plays crucial roles in cellular communication and signalling, with implications in spatial organisation, motility and differentiation. Similarly, the presence of an ECM-like extracellular polymeric substance is known to support and protect bacterial and fungal multicellular aggregates, such as biofilms or colonies. However, the roles and composition of this microbial ECM are still poorly understood.

**Results:**

This work presents a protocol to produce *S. cerevisiae* and *C. albicans* ECM in an equally highly reproducible manner. Additionally, methodologies for the extraction and fractionation into protein and glycosidic analytical pure fractions were improved. These were subjected to analytical procedures, respectively SDS-PAGE, 2-DE, MALDI-TOF-MS and LC-MS/MS, and DAE and FPLC. Additional chemical methods were also used to test for uronic acids and sulphation.

**Conclusions:**

The methodologies hereby presented were equally efficiently applied to extract high amounts of ECM material from *S. cerevisiae* and *C. albicans* mats, therefore showing their robustness and reproducibility for yECM molecular and structural characterization. yECM from *S. cerevisiae* and *C. albicans* displayed a different proteome and glycoside fractions. *S. cerevisiae* yECM presented two well-defined polysaccharides with different mass/charge, and *C. albicans* ECM presented a single different one. The chemical methods further suggested the presence of uronic acids, and chemical modification, possibly through sulphate substitution.

All taken, the procedures herein described present the first sensible and concise approach to the molecular and chemical characterisation of the yeast ECM, opening the way to the in-depth study of the microbe multicellular aggregates structure and life-style.

## Background

In multicellular organisms, communication between cells is essential, and is profoundly influenced by the extracellular matrix (ECM) components. This scaffolding structure coordinates the biochemical reactions of the different types of cells within the tissues and organs [1]. The mammalian ECM is composed by a wide array of functional molecules, biochemically and biophysically diverse, including proteins, glycosaminoglycans and proteoglycans. The glycosaminoglycans (GAGs) are some of the most dominant ECM components, greatly influencing the cellular behaviour. These highly charged polysaccharides, frequently sulphated, can be found covalently attached to protein cores, forming proteoglycans (PGs), which regulate the GAG distribution and turnover [2]. A high molecular diversity arises from the different combinations of PGs protein cores with one or more types of GAGs chain. These are responsible for the wide variety of biological roles, including structural scaffolding, signalling and growth factor storage [3].

Bacteria and fungi are able to form multicellular three-dimensional communities, such as stalks [4,5], mats/biofilms [6,7] and colonies [8-10]. These are supported and protected by an extracellular polymeric substance, or ECM, which originates from cellular synthesis and secretion processes, as well as from the lysis of embedded cells. When these microbial multicellular communities developed in natural environments, their ECM components account as well with contributions from the surrounding environment, for example from an infection host [11-13].

The ECM from *Saccharomyces cerevisiae* colonies revealed the presence of proteins [14], some of which highly glycosylated [15], which remain unidentified. In *Candida* ECM biofilms, several studies also reported the presence of proteins, as well as polysaccharides and DNA [16-18]. Importantly, an exopolysaccharide composed of α-*D*-glucose and β-*D*-glucose, α-*D*-mannose, α-*L*-rhamnose and N-acetyl glucosamine was identified in the ECM from a *C. albicans* biofilm recovered from an infected intrauterine device [12]. Additionally, the ECM-like substance from flocs of *S. cerevisise,* overexpressing *FLO1,* were shown to be composed of glucose and branched mannose [19]. Overall, the major players of the yeast colonies and biofilms remain unknown. Therefore, the identification of the molecules composing yeast ECM will contribute for the future understanding of cell-cell communication and other multicellular aggregation processes, namely quorum sensing.

This work provides the foundations for the detailed identification of yeast ECM (yECM) components, presenting the development of a protocol to produce yECM, which robustness was challenged through inter-species reproducibility. Subsequently, methodologies for yECM extraction and fractionation were optimized, providing unprecedented analytical-grade protein and sugar fractions for chromatographic assessment. These methodological advances will open the way to future research on the processes and players of eukaryotic multicellular life-style.

## Results and discussion

### Overlay development and ECM extraction

Molecular and structural assessment of yECM components requires a high amount of cells-free homogenous material, chemically purified into fractions proper for applying analytical techniques. Therefore, a critical step is the reproducible growth of yeast onto large multicellular aggregates without risking large biological heterogeneity between cells, either in very diverse metabolic states and/or different life cycle phases. *S. cerevisiae* growth onto biofilms is not common. Previous studies showed that the yECM recovered from a *S. cerevisiae* single colony, or a set of some colonies, was not enough for the in-depth analysis of its components [14]. The development of giant colonies, originated from a drop with several thousand cells, was the most common approach to overcome this obstacle [7,20]. We took this concept one step further and inoculated enough cells to produce a whole-plate three-dimensional overlay. The method was tested with wild type strains of *S. cerevisiae* and *C. albicans.*

**Figure 1:**
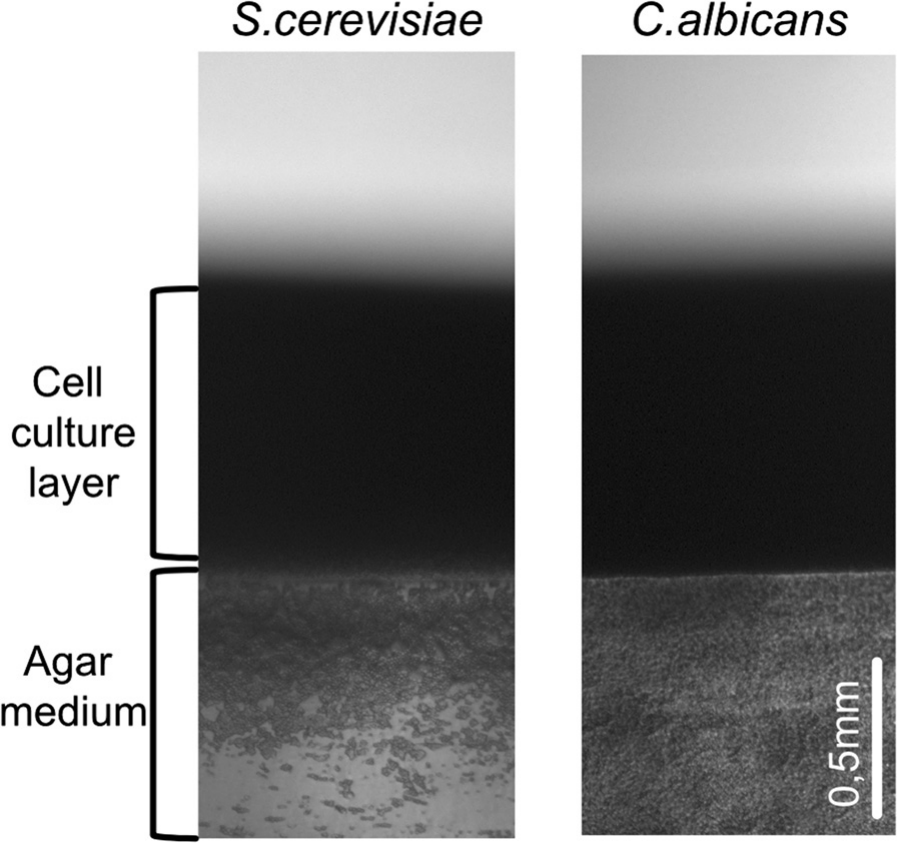
**Overlays of** ***S. cerevisiae*** **and** ***C. albicans*** **cultures.** Longitudinal cuts of overlays of cultures of *S. cerevisiae* and *C. albicans* grown for 7 and 5 days, respectively, on YPD at 30°C. Micrographs were obtained with a Leica Microsystems DM-RB fluorescence microscope and uEye digital camera.

Cells collected from a single colony were grown overnight until exponential growth phase, and these fully active cells were used to inoculate homogeneously the whole surface of the YPDa plates. The plates were firstly dried under a sterile air flow until the *inoculum* was properly absorbed and then incubated at 30°C. The cell growth and overlay development was followed until the so-called “mature overlay” presented a high quantity of biomass. This happened as a result of 7 days of growth for the case of *S. cerevisiae*, and 5 days growth in the case of *C. albicans*, consistently with the latter being a faster grower. Longitudinal slices of these cultures are shown in Figure 1. The further development of the culture into more days of incubation was assessed, yet resulted in an increased accumulation of dead cells (lysis) in the interior of the aggregate as monitored by flow cytometry (not shown), which is in accordance with previous works [13,21]. Multicellular aggregates in their innate environment, during development and maturation, will inevitably contain intracellular material deriving from cellular debris originated from cell death [13,18,21], which may eventually play some role in the aggregate biological properties. Nevertheless, the method hereby devised aimed at avoiding as far as possible this “contamination” so to assess the basic components of yECM.

The biomass from the mature overlay was carefully collected through washes with PBS buffer. Considering the interference of NaCl in several biochemical analysis techniques, namely 2DE and NMR, and in order to maximize the utilisation of yECM samples by a large variety of techniques, the amount of NaCl in the PBS buffer was reduced to 100 mM. The cellular suspension was very gently placed into a roller incubator to promote the solubilisation of the several molecules present in the intercellular space, followed by centrifugation at 15.000 rpm to induce the deposition of the cells and insoluble molecules. The supernatant was further cleaned by filtration, reducing the possibility of drag intracellular material of the next steps. The filtered supernatant was subsequently lyophilized to enable the concentration of the sample and the resuspension in the appropriate buffers for the following phases of the protocol.

The above-mentioned procedures allowed to retrieve the *S. cerevisiae* and *C. albicans* overlay biofilm-like yECM in high amounts, crucial for their molecular and structural-characterisation, while ensuring reproducibility between samples, and a low level of putative sample contamination with intracellular contents.

### ECM purification and analysis

#### Proteins

Few studies addressed the proteins present in the extracellular space of microbial multicellular communities, and most of such studies focus on liquid cultures [22-24]. In *S. cerevisiae* colonies*,* proteins and glycoproteins from the extracellular environment were briefly assessed [14,20]. The hereby-developed protocol recovered the proteins present in the yECM and allowed a proteomics approach.

The samples intended for the precipitation and recovery of proteins were collected in the presence of a cocktail of protease inhibitors (see Methods), precluding the action of proteases, remodelling enzymes or others in yECM. Precipitation was done using the chloroform/methanol protocol by Wessel and Flugge [25]. This protocol was chosen to avoid the presence of salt and other contaminants detrimental to the protein analysis and identification procedures. A high amount of proteins was recovered, based on the different solubility in diverse solvents. The current protocol avoids the addition of compounds such as TCA (trichloroacetic acid) or DOC (2,5-dimethoxy-4-chloroamphetamine) that compromise the downstream analysis. Furthermore, this protocol in comparison with TCA-based protocols, presents the extra advantage of not promoting the formation of an insoluble pellet, therefore maximizing protein recovery. Importantly, this method also allows the resuspension of the proteins directly in the sample buffer, after evaporation of the solvents.

**Figure 2:**
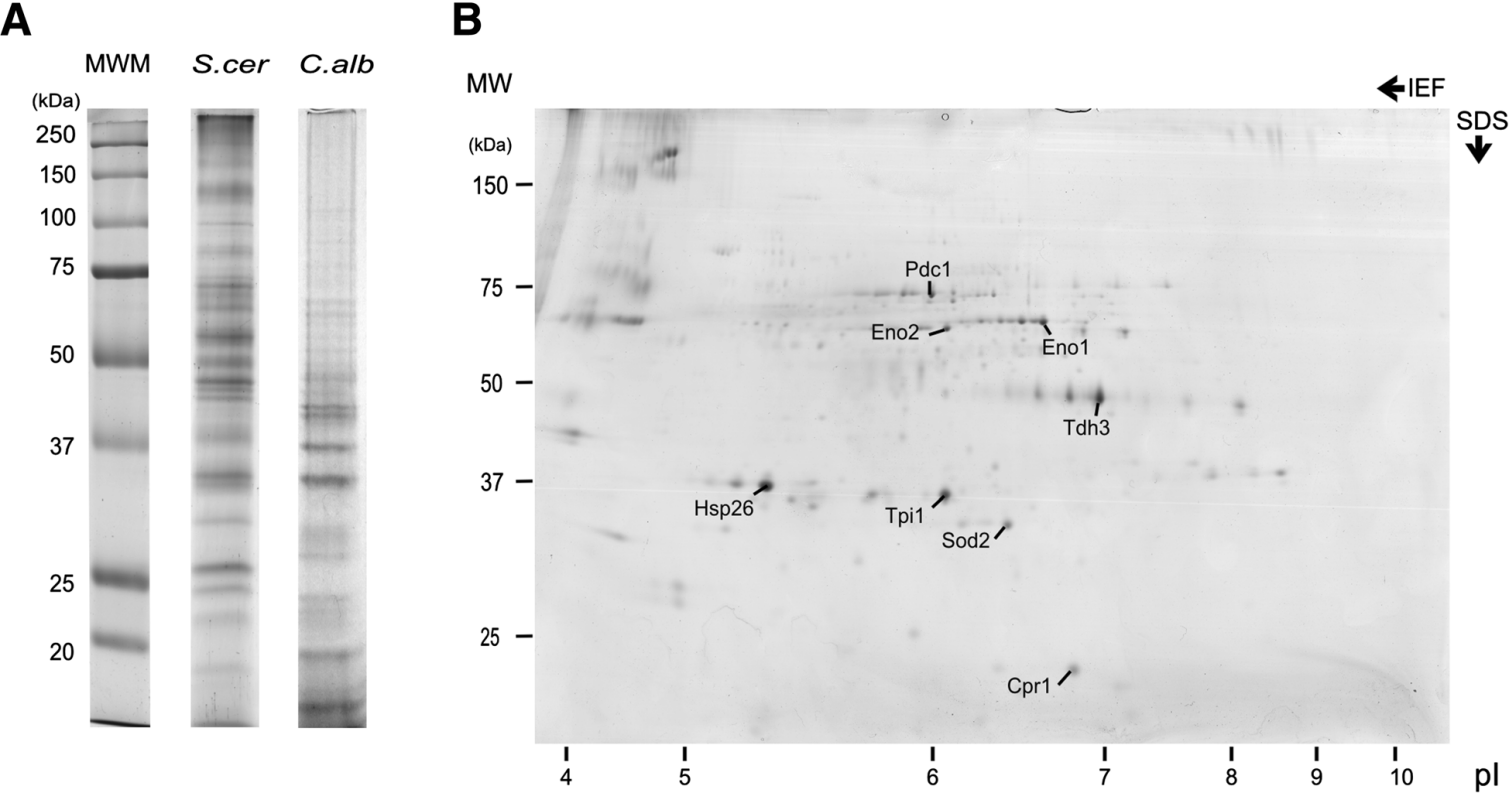
**SDS-PAGE and 2DE separation of yECM proteins. (A)** SDS-PAGE of the ECM proteins from *S. cerevisiae* and *C. albicans*. The methodology was capable of recover the extracellular proteins from two yeast species and detect the differences in the ECM composition. **(B)** 2DE of the *S. cerevisiae* strain ECM proteins. The 2DE allowed the identification of proteins present in the yECM (see Methods).

The isoelectric focusing (IEF)-compatible buffer mentioned in Materials and Methods presents a high protein solubilisation capacity and is compatible with the most common protein quantification methods, namely with Bradford quantification [26]. Moreover, this sample buffer is well suited for both SDS-PAGE and 2DE (Figure 2). This guaranties that the same sample can be analysed by both techniques, without resorting to different buffers that may not solubilize with the same efficiency.

The combination of all these procedures results in a highly reproducible methodology, which allows the analysis of ECM from different yeast strains with similar success (Figure 2A). Importantly, the protein samples obtained through such methodology are pure enough to be analysed by 2DE, known for its sensitivity to detergents, salts, and other molecules that interfere with the proteins’ isoelectric point. Besides, those samples can further be subjected to more sensitive techniques as 2D-DIGE or mass spectrometry. Figure 2B displays the 2DE gel of *S. cerevisiae* yECM protein sample. The two dimension separation of the proteins also allowed the unambiguous identification of several proteins present in the *S. cerevisiae* ECM, the glycolytic Tdh3 (glyceraldehyde-3-phosphate dehydrogenase), Tpi1 (triose phosphate isomerase), Pdc1 (pyruvate decarboxylase 1), Eno1/2 (enolase 1 and 2), Sod2 (superoxide dismutase 2), Hsp26 (heatshock protein 26), and Cpr1 (cytoplasmatic peptidyl-prolyl cis-trans isomerase) (Figure 2B). Some of these proteins were previously described in the extracellular environment by several authors (reviewed by [27]). Namely, Tdh3 was found overexpressed in the ECM of *C. albicans* biofilms [28], and enolase has been reported externally located in both yeasts and mammalian cells [23]. In yeasts, proteins found using the present procedures may derive from the outer layers of the cell wall as previously suggested (reviewed by [27]). Their location *in vivo* has though to be secured by loose attachment, since the present procedures preclude the extraction of covalently linked cell wall materials that require harsh methods to be extracted [29,30]. The identification of all the proteins present in yECM, will be possible analysing all the spots present in the 2DE, or using a high throughput technique, as liquid chromatography coupled with tandem mass spectrometry (LC-MS/MS).

### Polysaccharides

Similarly to what occurs with the proteins, little information is available on the polysaccharides of yECM, other than the presence of a few glycoproteins in *S. cerevisiae* colonies, and the identity of some components of *C. albicans* biofilms [12,14,20]. The sugar fraction of yECM was therefore collected and analysed, for which the procedures widely used to extract the high Eukaryotes ECM were adjusted [31-33]. The samples were subjected to the action of the broad range protease papain, eliminating the proteins and releasing polysaccharides that might be attached to them. This reaction was performed overnight to warrant complete digestion. After protein separation by centrifugation, the samples containing solubilized polysaccharides were treated with ethanol overnight at 4°C. Ethanol, disrupts the hydration of the polysaccharide and the charged ions, and when combined with low temperatures, maximizes the precipitation of both neutral and acidic polysaccharides. The utilisation of ethanol in this step is also beneficial because it is easily removable by evaporation. The precipitated polysaccharides can be dissolved, without difficulty, in deionized water or other required solution for the subsequent analysis.

**Figure 3:**
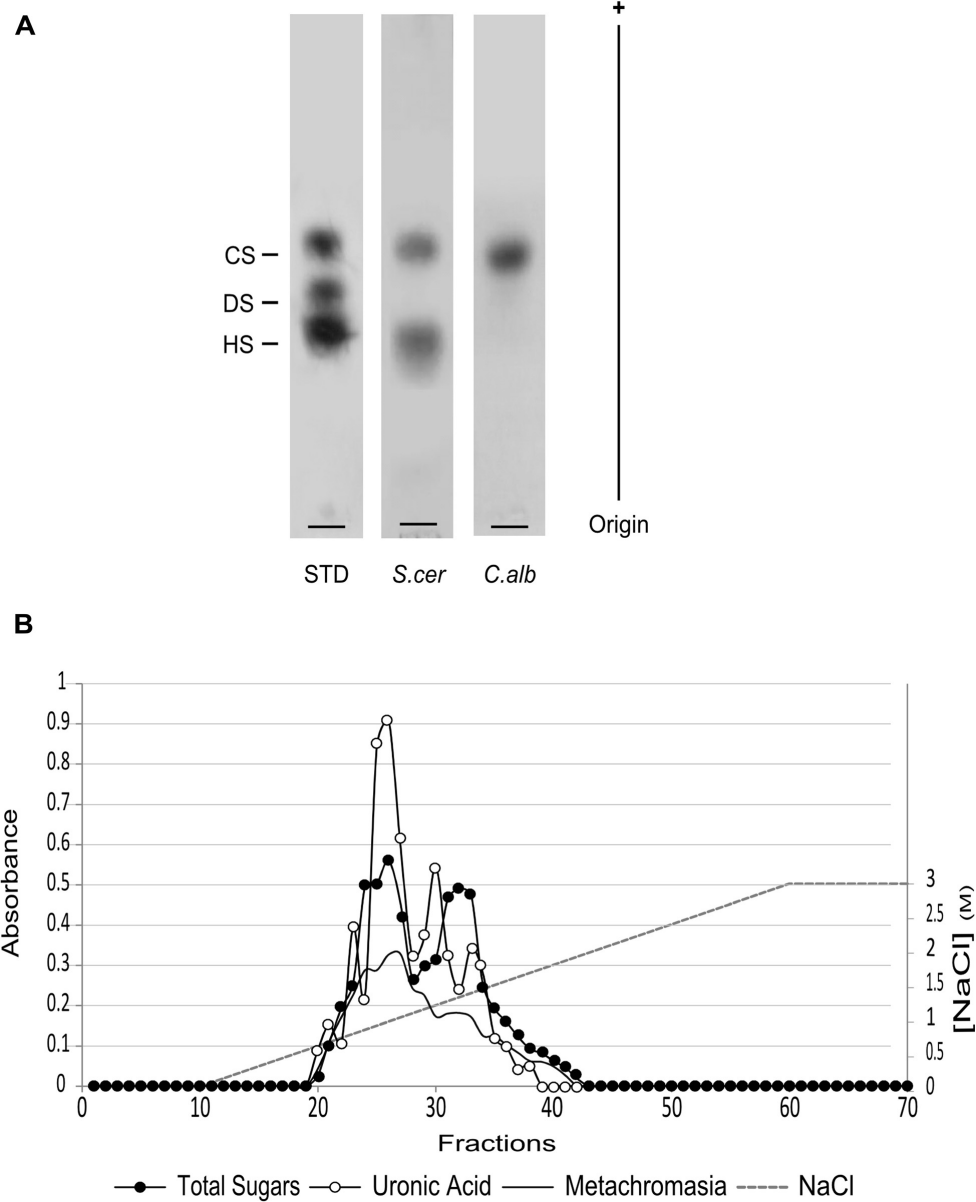
**Electrophoretic profiles and chromatographic fractionation of yECM polysaccharides. (A)** Diaminopropane agarose electrophoresis of the ECM polysaccharides from *S. cerevisiae* and *C. albicans*. The methodology was efficient in detecting differences between the two species yECM glycosidic fraction. STD – GAG standards (see and Methods). **(B)** Anionic exchange fractionation of *S. cerevisiae* ECM sample. Fractions were analysed for the presence of total sugars (dark circles), which indicate the presence of at least two different compounds. Fractions were also analysed for uronic acids (white circles) and metachromasia (black line).

The *S. cerevisiae* and *C. albicans* yECM polysaccharides recovered by this procedure were evaluated using 1,3-diaminopropane acetate agarose electrophoresis (DAE) (Figure 3A). This technique allows the separation of compounds with different degrees of chemical substitution, and is commonly used to separate sulphated polysaccharides [34]. *S. cerevisiae* presented two differently migrating compounds, whereas using *C. albicans* only one compound was detected. This suggests that the yECM composition is different from species to species. Importantly, the methodology proved capable of detecting those differences, showing its appropriateness for the study of yECM.

*S. cerevisiae* ECM polysaccharides were further analysed by fast performance liquid chromatography (FPLC) (Figure 3B). Identically to DAE, this technique also separates polysaccharides through the total charge-to-mass ratio. In this way, fractions of *S. cerevisiae* were collected and tested for (1) total sugars through reaction using sulphuric acid:phenol reaction [35], (2) uronic acids, through carbazole method [36], and (3) possible chemical substitution, analysing the metachromatic shift of 1,9-dimethylmethylene blue (DMMB) [37] (Figure 3B). In total accordance with the DAE results, the *S. cerevisiae* FPLC profile shows two major peaks (dark circles), indicating the existence of at least two different polysaccharide species. The presence of some kind of uronic acid in their composition (white circles), is suggested by carbazole staining, and chemical substitution by the presence of metachromasia (black line). Uronic acids are very common in extracellular polysaccharides, both in microorganisms and higher Eukaryotes [38,39]. Conversely, sulphation is the most frequent chemical substitution of higher Eukaryotes ECM polysaccharides [2].

## Conclusions

The present work provides the foundations for the detailed identification of yECM components and the understanding of its biological functions. A novel and simple protocol to produce yECM from overlays/mats multicellular aggregates was developed. Its robustness was challenged through inter-species reproducibility, using *S. cerevisiae* and *C. albicans*. The overlay culture mimicry of a multicellular aggregate warrants the future study of yECM development and regulation, through easy modulation of the microbial culture conditions or yeast genetic background. Moreover, methodologies for yECM extraction and fractionation were optimized, providing analytical-grade protein and sugar fractions, in view of its further assessment by powerful chromatographic analysis, as far as NMR. The procedures in this work consist in the first sensible and easy to use approach to support molecular and chemical characterisation of the yECM, opening the way to the in-depth study of the microbe multicellular aggregates structure and life-style.

## Methods

### Strains and media

Yeast strains used in this work were *S. cerevisiae* W303 (*MATa leu2-3 leu2-112 ura3-1 trp1-1 his3-11 his3-15 ade2-1 can1-100*) [40] and *C. albicans* BWP17 (*ura3::imm434/ura3::imm434 iro1/iro1::imm434 his1::hisG/his1::hisG arg4/arg4*) [41]. Cells were grown on rich medium, YPD (yeast extract 1%; peptone 2%; glucose 2%; adenine hemisulphate 0.005%) on an orbital shaker, 200 rpm, at 30°C. Growth was monitored by optical density (OD) at 600 nm. Solid growth was performed in YPD supplemented with agarose (2%, w/v) - YPDa. All ingredients percentages are presented as weight per volume units.

### Overlay development

The development of a three-dimensional overlay was preceded by batch cultures grown overnight on liquid YPD, with an air:liquid ratio of 2:1. An inoculum of 1.5 ml batch cultures at OD_600_ = 1 was spread in YPDa plates, using circular movements to ensure an even distribution. The plates were left to completely absorb the inoculum under a laminar flow. The dried plates were incubated at 30°C and the overlay was allowed to develop for 7 days (*S. cerevisiae*) and for 5 days (*C. albicans*). Longitudinal cuts of the agar and cellular overlay were placed onto a slide and observed under a Leica Microsystems DM-RB fluorescence microscope with a magnifying lens N PLAN 5x/0.11 (Leica P/N 506029), in bright field. Images were acquired through an IDS uEye USB 2.0 camera and processed with uEye Cockpit Application Software.

### Matrix extraction

The cellular overlay was carefully collected into a 50 ml Falcon tube with PBS buffer (NaCl 100 mM; KCl 2.7 mM; Na_2_HPO_4_.2H_2_O 10 mM; KH_2_PO_4_ 2.0 mM; pH 7.4). The collected biomass was incubated for 10 minutes with constant rotation in a tube roller (SRT1; Stuart, Staffordshire, UK) to allow the complete suspension of proteins and polysaccharides. The samples intended for protein purification and analysis, were supplemented with a protease inhibitor cocktail (PMSF 0.2 μg/ml; Aprotinin 0.32 μg/ml; Pepstatin 1 μg/ml; Leupeptin 1 μg/ml). The suspension, containing cells and extracellular matrix, was then centrifuged for 10 minutes at 15,000 rpm and 4°C in a Sigma 4-16 K centrifuge (Sigma, Osterode, Germany) to promote the deposition of the insoluble material. The supernatant was collected, filtered through a 0.45 μm membrane and freeze-dried (Christ Alpha 2–4 Christ LDC-1 m, B. Braun). Membrane integrity was assessed by cytometry as described before [42]. Briefly, cells were harvested and added 4 μg/ml propidium iodide (Sigma). After 10 min incubation in the dark at room temperature, the samples were analysed in an Epics® XL™ (Beckman Coulter) flow cytometer.

### ECM precipitation and recovery

Proteins - The lyophilized extract was resuspended in MilliQ water (just enough volume to completely solubilize the overlay components). The proteins were precipitated using the chloroform/methanol protocol [25]. The resulting pellet was left at room temperature to evaporate the remaining methanol, and then resuspended in isoelectric focusing IEF buffer (urea 7 M; thiourea 2 M; CHAPS 2%, w/v) [43], which is compatible with downstream analysis. Alternatively, Laemmli buffer (Tris–HCl 125 mM, pH 6.8; glycerol 20%, w/v; SDS 4%, w/v), modified through the further addition of 6 M urea and 1% w/v DTT– ML buffer, was used with similar efficiency for SDS-PAGE purposes. Protein was quantified with Bio-Rad Protein Assay (Bio-Rad, Richmond, CA, USA) as recommended by the manufacturer.

Polysaccharides - The lyophilized supernatant was resuspended in digesting buffer (NaOAc 100 mM; EDTA 5 mM; cysteine 5 mM; pH 5.5) in a proportion of 20 ml per gram of dry weight. Double-crystalized papain (Merck, Darmstadt) was added to the mixture (10 mg/ml) and incubated at 60°C overnight. The mixture was centrifuged (3,000 rpm for 10 minutes at room temperature) and the clear supernatant was collected. The recovery of yECM polysaccharides was achieved through ethanol precipitation. Three volumes of ethanol (95-99%, v/v) were added to the supernatant and incubated overnight at 4°C. The precipitate was collected by centrifugation, 10 minutes at 3,000 rpm, and left at room temperature until total evaporation of the residual ethanol. An aliquot of the pellet was resuspended in deionised water and was evaluated for (i) total sugar content, by the reaction of phenol-sulphuric acid with neutral sugars [25], (ii) the presence of hexuronic acids, through carbazole method [26], and (iii) chemical substitution by metachromatic shift of 1,9-dimethylmethylene blue (DMMB) [27].

### ECM analysis

#### Proteins

One and two dimensions electrophoresis - The analysis of the protein profiles was performed by one-dimensional electrophoretic separation under denaturing conditions (SDS-PAGE) in a Mini PROTEAN® 3 Cell apparatus (Bio-Rad) and by 2DE in a DALTSix system (GE Healthcare).

The SDS-PAGE was carried out in a 1.5 mm thick polyacrylamide gel, with a 4% stacking gel and a 10% resolving gel. The sample (5 μg) was brought up to 10 μl with MilliQ water and mixed with loading buffer, Laemmli 5X [44]. The mixture was boiled for 5 minutes and then transferred to ice, for at least 5 minutes. The sample was loaded and the electrophoresis was run at 100 V, for 2 hours or until the migration front reached the end of the gel.

For the 2DE, the samples were primarily submitted to IEF - first dimension - and then run according to molecular weight in a homogeneous polyacrylamide gel (10% of total polyacrylamide and 2.6% of bis-acrylamide - 10% T, 2.6% C) - second dimension. The IEF dry strips (24 cm, pH 3-11NL) were incubated in Rehydration buffer (urea 8 M; thiourea 2 M; CHAPS 4%, w/v; IPGphor buffer pH 3–11 2%, w/v; DeStreak 1.5%, w/v) for at least 8 hours. The sample, mixed with a 2X IPGphor buffer pH 3-11/DTT solution, was applied by cup loading. The IEF was performed at 20°C in an Ettan IPGphor 3 apparatus (GE Healthcare), according to the following program: 120 V for 1 h; 500 V for 2 h; 500–1000 V in gradient for 2 h; 1000–5000 V in gradient for 6 h; 5000 V for 10 h. After this, strips were equilibrated first in reducing solution (urea 6 M; Tris–HCl pH 6.8 50 mM; glycerol 30%. v/v; SDS 2%, w/v; dithiothreitol 2%, w/v) for 12 minutes, and then 5 minutes in alkylating solution (Urea 6 M; Tris–HCl pH 6.8 50 mM; glycerol 30%; v/v; SDS 2%, w/v; iodoacetamide 2.5%, w/v). Second dimension SDS-PAGE were run on homogeneous polyacrylamide gel (12% T, 2.6% C). Electrophoresis was carried out at 18°C, 1 W/gel for 18 hours, using Ettan DALTsix system (GE Healthcare). Both SDS-PAGE and 2DE gels were stained according to the Colloidal Coomassie Blue protocol [45,46], and scanned with a calibrated densitometer (Bio-Rad; Molecular Imager GS-800).

Protein Identification - The chosen spots from the 2DE gels were excised and in-gel digested [47]. Samples were reduced with 10 mM DTT, in ammonium bicarbonate (25 mM; pH 8.5), for 30 minutes at 56°C and subsequently alkylated with 55 mM iodoacetamide, in ammonium bicarbonate (25 mM; pH 8.5), for 20 minutes in the dark. Finally, samples were digested with 12.5 ng/μl of sequencing grade trypsin (Roche Molecular Biochemicals), in ammonium bicarbonate (25 mM; pH 8.5), overnight at 37°C.

The spots supernatants were collected and 1 μl was spotted onto a MALDI target plate and allowed to air-dry at room temperature. To each digested spot was added 0.5 μl of α̣-cyano-4-hydroxytranscinnamic acid matrix (Sigma; 3 mg/ml in acetonitrile 50%; v/v), and allowed again to air-dry at room temperature. MALDI-TOF MS analyses were performed in a MALDI-TOF/TOF mass spectrometer 4800 plus Proteomics Analyzer (Applied Biosystems. MDS Sciex, Toronto, Canada) and 4000 Series Explorer™v 3.5 Software (ABSciex). The instrument was operated in reflector mode, with an accelerating voltage of 20000 V. All mass spectra were internally calibrated using peptides from the autodigestion of the trypsin. MALDI-TOF MS analysis produces peptide mass fingerprints, which can be collected and represented as a list of monoisotopic molecular weights with a Signal to Noise ratio greater than 12. The suitable precursors for MS/MS sequencing analysis were selected and fragmentation was carried out using the Collision-induced dissociation on (atmospheric gas) 1 kV ion reflector mode and precursor mass Windows +/−4 Da. The plate model & default calibration were optimized for the MS-MS spectra processing. The search of peptides was performed in batch mode using GPS Explorer v3.5 software (ABSciex) with a licensed version 2.3 of MASCOT (www.matrixscience.com), using the NCBInr database (date: 08052012; 17919084 sequences; 6150218869 residues). The MASCOT search parameters were: (1) species: *S. cerevisiae*; (2) allowed number of missed cleavages: 1; (3) fixed modification: carbamidomethyl cysteine, (4) variable modifications: methionine oxidation; (5) peptide tolerance: ±50 ppm for PMF and 80 ppm for MS/MS searches; (6) MS/MS tolerance: ±0.3 Da and (7): peptide charge: +1. In all identified proteins, the probability score was greater than the one fixed by Mascot as being significant, that is, a p value under 0.05.

#### Polysaccharides

Electrophoretic analysis - ECM total polysaccharide extracts were submitted to agarose gel electrophoresis as described previously [48]. Briefly, about 1.5 μg of the sample (as of uronic acid content) was applied to a 0.5% agarose gel in a 1,3-diaminopropane acetate buffer (50 mM; pH 9.0), and run for 1 h at 100 V. As standard, a mixture of sulphated polysaccharides containing chondroitin sulphate, dermatan sulphate, and heparan sulphate (1.5 μg of uronic acid of each), was used. The polysaccharides were fixed with aqueous 0.1% cetyl-trimethylammonium bromide solution (Cetavlon®), the gel was allowed to dry, and stained with 0.1% toluidine blue in acetic acid/ethanol/water (0.1:5:5, v/v/v).

FPLC - An aliquot of the ethanol-precipitated pellet (≈20 mg) was resuspended in 1 ml MilliQ water, filtered through a syringe filter (0.22 μm) for analysis in a FPLC system (Pharmacia Biotech, Sweden). The filtered sample was applied to a Hitrap Q-XL column, equilibrated with elution buffer (Tris–HCl 20 mM; pH 8.6). The polysaccharides were eluted by a linear gradient of 0–3.0 M NaCl, at a flow rate of 0.50 ml/min, and fractions of 0.5 ml were collected. The total sugar content of the fractions was evaluated by the reaction of phenol-sulphuric acid with hexoses [35]. The presence of hexuronic acids was assessed through the carbazole method [36], and the possible chemical substitution, tested by the metachromatic shift of 1,9-dimethylmethylene blue (DMMB) [37].

## Abbreviations

ECM: Extracellular matrix
SDS-PAGE: Sodium dodecyl sulphate polyacrylamide gel electrophoresis
2-DE: Two-dimensional gel electrophoresis
MALDI-TOF-MS: Matrix-assisted laser desorption-ionization time-of-flight mass spectrometry
LC-MS/MS: Liquid chromatography coupled with tandem mass spectrometry
DAE: 1,3-Diaminopropane acetate agarose electrophoresis
FPLC: Fast performance liquid chromatography
GAGs: Glycosaminoglycans
PGs: Proteoglycans
yECM: Yeast extracellular matrix
NMR: Nuclear magnetic resonance
IEF: isoelectric focusing
2 DE-DIGE: Two-dimensional difference gel electrophoresis
DMMB: dimethylmethylene blue
